# Fluorescent bioassays for toxic metals in milk and yoghurt

**DOI:** 10.1186/1472-6750-12-76

**Published:** 2012-10-25

**Authors:** Mohammad Shohel Rana Siddiki, Shunsaku Ueda, Isamu Maeda

**Affiliations:** 1 United Graduate School of Agricultural Science, Tokyo University of Agriculture and Technology, 3-5-8 Saiwaicho, Fuchu 183-8509, Japan; 2 Faculty of Agriculture, Utsunomiya University, 350 Minemachi, Utsunomiya 321-8505, Japan

## Abstract

**Background:**

From a human health viewpoint, contaminated milk and its products could be a source of long-term exposure to toxic metals. Simple, inexpensive, and on-site assays would enable constant monitoring of their contents. Bioassays that can measure toxic metals in milk or yoghurt might reduce the risk. For this purpose, the green fluorescent protein (GFP)-tagged *trans* factors, ArsR-GFP and CadC-GFP, together with their *cis* elements were used to develop such bioassays.

**Results:**

ArsR-GFP or CadC-GFP, which binds either toxic metal or DNA fragment including *cis* element, was directly mixed with cow’s milk or yoghurt within a neutral pH range. The fluorescence of GFP, which is reflected by the association/dissociation ratio between *cis* element and *trans* factor, significantly changed with increasing externally added As (III) or Cd (II) whereas smaller responses to externally added Pb (II) and Zn (II) were found. Preparation and dilution of whey fraction at low pH were essential to intrinsic zinc quantification using CadC-GFP. Using the extraction procedure and bioassay, intrinsic Zn (II) concentrations ranging from 1.4 to 4.8 mg/l for milk brands and from 1.2 to 2.9 mg/kg for yoghurt brands were determined, which correlated to those determined using inductively coupled plasma atomic emission spectroscopy.

**Conclusions:**

GFP-tagged bacterial *trans* factors and *cis* elements can work in the neutralized whole composition and diluted whey fraction of milk and yoghurt. The feature of regulatory elements is advantageous for establishment of simple and rapid assays of toxic metals in dairy products.

## Background

Toxic metal contamination to foods causes major global health problems. Humans are exposed to toxic metals primarily from air, water, and food [1]. Pollution of foods with environmental toxic metals even in trace quantities has attracted considerable attention in the global era with rapid transportation. The simple and inexpensive monitoring of food pollution needs to be developed for reducing or eliminating the amounts of toxic elements into the environment. Milk and milk products provide good quality nutrients necessary for a strong healthy body and mind, and act as a primary source of nutrients in diets all around the world [2]. However, the presence of toxic elements in milk and milk products may create health problems especially for infants, school age children, and old people who consume large quantity of those products. Their presence in milk and its products is caused by different agricultural activities. Irrigation with toxic metal-contaminated water and use of pesticides, parasiticides, drugs, and environmental disinfectants to cows may result in toxic metal contamination in feeds [3], meat, and milk [4-6]. As the mammary glands are the most physiologically sensitive part of dairy cows, the input and output of toxic metals in these organisms are clearly reflected in the milk [7]. Heavy metals, specially cadmium, arsenic, zinc, and lead, are ubiquitously found in nature and, therefore, their contamination to milk and milk products must be considered [1]. Among these toxic metals, zinc is the most abundant one [8] and provided to humans. Although an adequate amount of zinc is physiologically important, exposure to the excess amount is harmful and toxic aspects of zinc arise [9]. Therefore, it becomes consumers’ benefits to monitor whether the zinc concentration in milk or milk products is adequate or not.

Analytical methods towards hazardous chemical compounds in environments and foods have attracted much attention. Flame atomic absorption spectrometry (FAAS), electrothermal atomic absorption spectrometry (ET-AAS), inductively coupled plasma atomic emission spectroscopy (ICP-AES), inductively coupled plasma mass spectrometry (ICP-MS), hydride generation coupled with atomic absorption spectroscopy (HG-AAS) or atomic fluorescence spectroscopy (HG-AFS), X-ray spectroscopy, spectrofluorimetry, spectrophotometry, and electroanalytical techniques have been commonly used for determination and quantification of metals [9]. However, such standard methods require expensive and bulky laboratory equipments, analytical expertise and sample transportation, and generate hazardous wastes [10,11]. Expensive and bulky HG-AAS and HG-AFS have limits of detection (LODs) in the microgram per kilogram range [12] although ICP-AES sensitivity can be improved by coupling to HG. Under certain circumstances, sensing approaches with low cost can compete with traditional analyses in South-East Asia where contaminations by massive arsenic or other heavy metals in water or foods occur. In this sense, biosensors that measure bioavailable fractions of toxic metal ion have attracted much attention. To compensate the weaknesses of traditional methods, the biosensors that can detect As (III) and Cd (II)/Pb (II)/Zn (II) in water have been developed using the *trans* factors/*cis* elements, ArsR/*ars* promoter−*ars* operator (*P*_ars_−*O*_ars_) and CadC/*cad* promoter−*cad* operator (*P*_cad_−*O*_cad_) [13]. ArsR, encoded by *arsR*, binds exclusively to either *P*_ars_*−O*_ars_ or As (III), and CadC, encoded by *cadC*, binds to either *P*_cad_*−O*_cad_ or Cd (II). The simple, inexpensive, and sensitive analysis of toxic metals in water was achieved by fusing ArsR or CadC to green fluorescent protein (ArsR-GFP or CadC-GFP). However, although different types of GFP-tagged biosensors have been developed for on-site determination of toxic metals in drinking water and soil extracts [13,14], these biosensors have not been tested for foods. Therefore, it remains unknown in milk and milk products whether these recombinant proteins keep their binding capabilities to metals or DNA and what amounts of toxic metals are quantified with previously established methods.

The aims of this study are to investigate responsiveness of *trans* factors that can bind to toxic metals or DNA in whole composition of cow’s milk and yoghurt as well as in whey fractions and to develop simple methods for determination of toxic metals in the dairy products by application of the GFP-tagged *trans* factors and immobilized *cis* elements.

## Results

### ArsR-GFP responds to externally added As (III)

**Figure 1:**
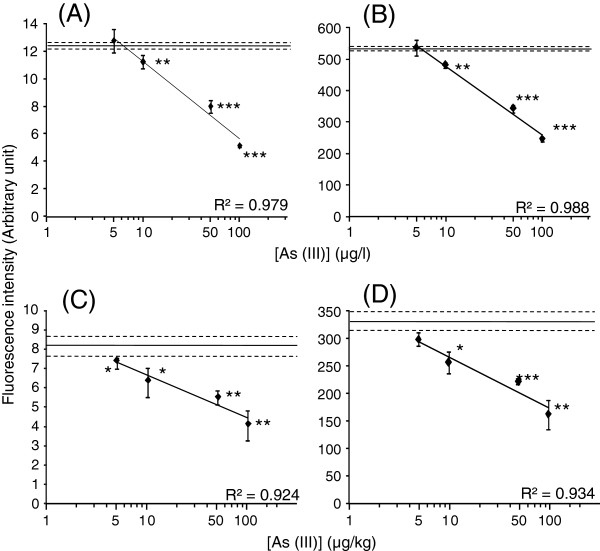
**Fluorescence values arose from the ArsR-GFP associated with *P***_**ars**_**−*O***_**ars**_**after incubation with As (III)-added milk (A and B) and yoghurt (C and D) measured by fluororeader (A and C) and fluorometer (B and D).** A solid line and two broken lines show mean ± SD of data obtained with milk or yoghurt without addition of As (III). Asterisk means statistical significance versus the milk or yoghurt without addition of As (III) (*P < 0.05, **P < 0.01, ***P < 0.001).

Only inorganic As (III) resulted in a decrease of fluorescence and no responses to inorganic As (V) and the organic forms of As such as methylarsonic acid, cacodylic acid, and arsenobetaine were observed [13,14]. Arsenic concentrations in milk were low and mostly in the form of trivalent inorganic arsenic [15]. Therefore, inorganic As (III) was taken under consideration in this study. Whole milk and yoghurt, to which As (III) was externally added, were fluorometrically tested with the assay using the separately prepared biosensor. The fluorescence response of ArsR-GFP to the As (III) within 40 min was analyzed in milk (Figure 1A and B) and yoghurt (Figure 1C and D) with a microplate fluororeader (Figure 1A and C) and a portable fluorometer (Figure 1B and D). The results showed that fluorescence was significantly decreased at As (III) concentrations of 10–100 μg/l in milk and 10–100 μg/kg in yoghurt. The LODs for As (III) were determined to be 10 μg/l in milk and 10 μg/kg in yoghurt. The fluorescence intensities were linearly decreased with the increase in As (III) concentrations in milk (R^2^ = 0.979 and 0.988) (Figure 1A and B) and yoghurt (R^2^ = 0.924 and 0.934) (Figure 1C and D). Using the separately prepared biosensors, the same LODs and working range for As (III) externally added to milk and yoghurt were reproduced (Additional file 1A and C). In measurement by ET-AAS, however, non-linearities were obtained between the As (III) concentrations added to milk or yoghurt and the absorbance values. Only 100 μg/kg As (III) was detected by ET-AAS (Table 1). The result shows that the specific protein-DNA and protein-metalloid interactions can be applied to quantification of the lower concentrations of As (III) in whole milk and yoghurt in comparison with ET-AAS.

**Table 1:** Analytical performance of the biosensors compared with those of ICP-AES and ET-AAS

	**Biosensors**				**Traditional analytical method**^**1**^			
	**LOD**		**Detection range**		**LOD**		**Detection range**	
	**Milk (μg/l)**	**Yoghurt (μg/kg)**	**Milk (mg/l)**	**Yoghurt (mg/kg)**	**Milk (μg/kg)**	**Yoghurt (μg/kg)**	**Milk (mg/kg)**	**Yoghurt (mg/kg)**
External As (III)	10		10-100		100		100	
External Cd (II)	5		5-100		5		5-100	
Intrinsic Zn (II)			1.4-4.8	1.2-2.9			1.4-2.4	2.5-4.7

^1^ET-AAS and ICP-AES were used for measurement of externally added As (III)/Cd (II) and intrinsic Zn (II), respectively.

### CadC-GFP responds to externally added Cd (II) but not to externally added Pb (II)

**Figure 2:**
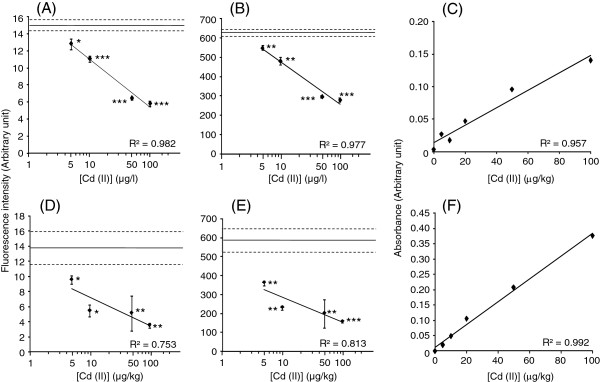
**Fluorescence values arose from the CadC-GFP associated with*P***_**cad**_**−*O***_**cad**_**after incubation with Cd (II)-added milk (A and B) and yoghurt (D and E) measured by fluororeader (A and D) and fluorometer (B and E).** Absorbance values of ET-AAS obtained with Cd (II)-added milk (**C**) and yoghurt (**F**). A solid line and two broken lines show mean ± SD of data obtained with milk or yoghurt without addition of Cd (II). Asterisk means statistical significance versus the milk or yoghurt without addition of Cd (II) (*P < 0.05, **P < 0.01, ***P < 0.001).

**Figure 3:**
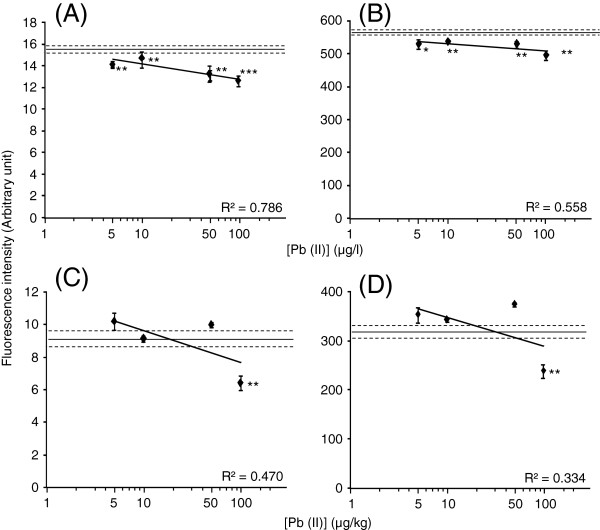
**Fluorescence values arose from the CadC-GFP associated with *P***_**cad**_**−*O***_**cad**_**after incubation with Pb (II)-added milk (A and B) and yoghurt (C and D) measured by fluororeader (A and C) and fluorometer (B and D).** A solid line and two broken lines show mean ± SD of data obtained with milk or yoghurt without addition of Pb (II). Asterisk means statistical significance versus the milk or yoghurt t without addition of Pb (II) (*P < 0.05, **P < 0.01, ***P < 0.001).

Whole milk and yoghurt, to which Cd (II) or Pb (II) was externally added, were also tested with the separately prepared biosensor. Responses of CadC-GFP were analyzed in whole milk and yoghurt using a fluororeader and a portable fluorometer. CadC-GFP responded to Cd (II) dose-dependently at concentrations of 5–100 μg/l in whole milk and 5–100 μg/kg in whole yoghurt when the fluorescence was measured by fluororeader and fluorometer (Figure 2). The LODs were 5 μg/l in whole milk and 5 μg/kg in whole yoghurt. The fluorescence was linearly decreased with the increase in Cd (II) concentrations in milk (R^2^ = 0.982 and 0.977) (Figure 2A and B) and yoghurt (R^2^ = 0.753 and 0.813) (Figure 2D and E). In measurement by ET-AAS, the linearity was obtained within a range of 0–100 μg/kg for milk (R^2^ = 0.957) and yoghurt (R^2^ = 0.992) (Figure 2C and F). The result shows that although the lower linearity is disadvantageous, almost same performance in terms of LOD as in ET-AAS is available in the fluorescent bioassay for Cd (II) in milk and yoghurt. Using the separately prepared biosensors, the same LODs and working range for Cd (II) in milk and yoghurt were reproduced (Additional file 1B and D). On the other hand, the fluorescent intensity of CadC-GFP significantly decreased at 5 μg/l Pb (II) in milk and 100 μg/kg Pb (II) in yoghurt. However, lower reduction of fluorescence intensity and lower linearity of the response were found within the tested range (Figure 3). Therefore, CadC-GFP might not be suitable for measurement of Pb (II) using whole products. The fluorescence decreases at each concentration were more marked in Cd (II) than in Pb (II) in the assays.

### CadC-GFP responds to Zn (II) extracted into whey fractions but not to Zn (II) in whole composition

**Figure 4:**
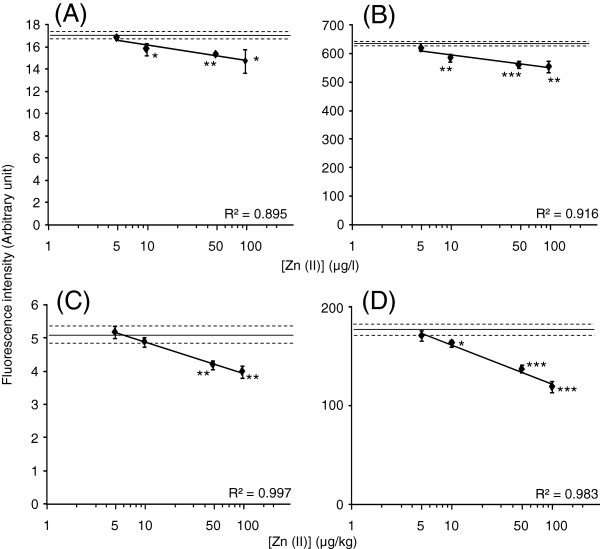
**Fluorescence values arose from the CadC-GFP associated with *P***_**cad**_**−*O***_**cad**_**after incubation with Zn (II)-added milk (A and B) and yoghurt (C and D) measured by fluororeader (A and C) and fluorometer (B and D).** A solid line and two broken lines show mean ± SD of data obtained with milk or yoghurt without addition of Zn (II). Asterisk means statistical significance versus the milk or yoghurt t without addition of Zn (II) (*P < 0.05, **P < 0.01, ***P < 0.001).

Whole milk and yoghurt, to which Zn (II) was externally added, were tested with the separately prepared biosensor. The significant responses of CadC-GFP to externally added Zn (II) were observed at 10 μg/l for whole milk (Figure 4A and B) and 50 or 10 μg/kg for whole yoghurt (Figure 4C and D). The linearity was obtained within a range of 5–100 μg/l for milk (R^2^ = 0.895 and 0.916) and within a range of 5–100 μg/kg for yoghurt (R^2^ = 0.997 and 0.983). However, as observed in the fluorescence responses to externally added Pb (II), low reduction of fluorescence intensity was found in response to externally added Zn (II) concentrations in milk and yoghurt. It has been reported that zinc in milk usually binds to the low molecular weight ligands as citrate and amino acids, and to proteins such as casein, α-lactalbumin, and lactoferrin [16,17]. It was expected that externally added Pb (II) or Zn (II) in whole milk and yoghurt might bind to the low molecular weight ligands or the milk proteins strongly. It has been reported that lowering pH of cow’s milk changed zinc and protein distribution and resulted in the shift of zinc from pellet (casein) to whey [16]. Therefore, pre-treatments for milk and yoghurt were considered. Firstly, pH of whole milk was lowered to 4.6 or below to change zinc and protein distribution and extract zinc from pellet into whey. Secondly, in order to reduce concentrations of substances in the whey fractions of yoghurt or acid-treated milk, 100-times dilution was performed with sterilized ultrapure water. The previous analysis concerning the CadC-GFP specificity revealed the enhancement of its background fluorescence by Ca [13]. Milk or yoghurt usually contains abundant elements such as Ca, P, Mg, Na, and Zn. Therefore, an excess amount of Ca (II) (10 mg/l) was externally added to Zn (II) standard solutions so that the effect of intrinsic Ca (II) in the diluted whey fractions on the background fluorescence was eliminated. The pH of standard solutions was adjusted to an average pH of tested whey fractions. Fluorescence intensities of CadC-GFP bound to *P*_cad_−*O*_cad_ after incubation with whey fractions from different brands of milk (Additional file 2a-c) and yoghurt (Additional file 2d-g) were measured using a portable fluorometer. The fluorescence intensities decreased in the separately prepared biosensor with increasing concentration of Zn (II) in the standard solutions (Additional file 2). This can be explained by an assumption that, in this assay, the fluorescence arises from the CadC-GFP associated with *P*_cad_−*O*_cad_ and the association constant between CadC-GFP and *P*_cad_−*O*_cad_ decreases in the presence of Zn (II). Besides this, CadC-GFP also responded to whey fractions derived from different brands of milk (Additional file 3a-c) and yoghurt (Additional file 3d and e) using the solid phase biosensor. Fluorescence intensities increased in response to Zn (II) in the standard solutions (Additional file 3). In this assay, the fluorescence arises from the CadC-GFP dissociated from *P*_cad_−*O*_cad_ and the dissociation constant between CadC-GFP and *P*_cad_−*O*_cad_ might increase in response to Zn (II) [14]. Measurement of Zn (II) concentrations in the whey fractions was repeated in different batches (Additional files 2 and 3). The concentrations of different brand of milk and yoghurt determined using the separately prepared biosensor varied from 1.5 to 4.8 mg/l and 1.8 to 2.9 mg/kg, respectively. When those were determined using the solid phase biosensor, 1.4 to 3.3 mg/l for milk and 1.2 to 2.5 mg/kg for yoghurt were obtained. These Zn (II) concentrations overlapped with the reported ranges of zinc content, which varied from 0.29 to 4.96 μg/g in raw bovine milk [5] and from 2.19 to 4.85 μg/g in yoghurt [18]. The result suggests that the pre-treatments and fluorescence measurements are adequate and reproducible in determination of Zn (II) concentrations.

### Correlation between zinc contents measured with the bioassays and ICP-AES

**Figure 5:**
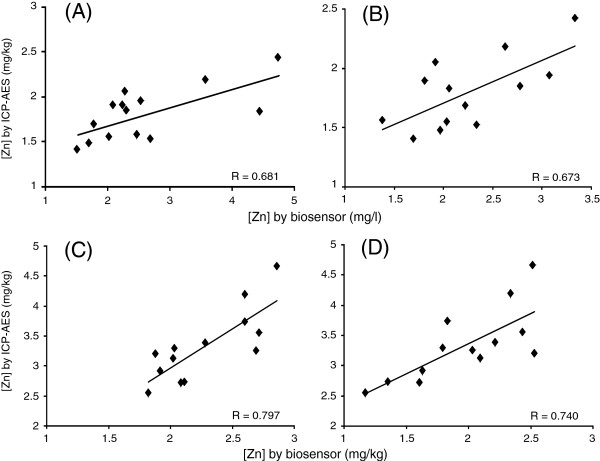
**Correlations between Zn (II) concentrations in the whey fractions prepared from milk (A and B) or yoghurt (C and D) and zinc concentrations in whole milk or yoghurt determined with ICP-AES.** The separately prepared biosensor (**A** and **C**) and solid phase biosensor (**B** and **D**) were used. The correlation coefficient (R) is shown in each panel.

The Zn (II) contents were determined towards the whey fractions in the bioassays. In order to evaluate whether or not the bioassays are available as simple and on-site protocols to measure zinc content in milk or yoghurt, zinc in whole milk or yoghurt was quantified with a standard protocol, ICP-AES. Then, correlations between the Zn (II) contents in whey fractions and the zinc contents in whole products were evaluated. Positive and linear correlations were found in the separately prepared biosensor for milk (Figure 5A) and yoghurt (Figure 5C). When bioassay was performed using the solid phase biosensor, the correlation coefficients for milk and yoghurt were smaller than those obtained with the separately prepared biosensor (Figure 5B and D). Although, the data obtained with biosensor and ICP-AES were not highly correlated, those were plotted within narrow concentration ranges of 1.3 to 4.8 mg/l or mg/kg for milk and 1.1 to 4.7 mg/kg for yoghurt. Therefore, both types of biosensor could detect an unexpected or abnormal value in routine monitoring of Zn (II) for milk and yoghurt with a simple protocol and a handheld device.

## Discussion

The bioassays to quantify toxic metals can even compensate with expensive instrumental analyses such as AAS, ICP, and AFS. For traditional analytical techniques such as ICP-AES and ET-AAS, dry mineralization or microwave-induced combustion methods are generally essential for quantitative extraction of toxic metals from samples. Contrary to necessity of these time-consuming pre-treatments, toxic metals in milk and yoghurt could be measured directly or only with preparation of whey and dilution in the fluorescent bioassays. The simple pre-treatment for the fluorescent bioassays was required to prepare transparent samples because the samples were loaded into the solid phase biosensors and provided directly to fluorescence measurement. It has been reported that casein was precipitated at pH 4.6 and about 90% of the zinc content and 95% of the citrate content identified as zinc citrate were released into the whey fraction [19]. This knowledge supports the results obtained in this study that zinc concentrations in whey fractions measured by biosensors correlated with those in whole milk measured by ICP-AES. The analytical performances of the biosensors, ICP-AES, and ET-AAS were summarized (Table 1). The reported ranges of arsenic, cadmium, and zinc are 0.001-0.15, 0.070-0.112, and 3.001-3.940 mg/kg in milk, and 0.01-0.35, 0.059, and 2.638 mg/kg in yoghurt [8,12]. Using the bioassays developed in this study, 0.01-0.1 mg/kg As (III), 0.005-0.1 mg/kg Cd (II) could be quantified in milk and yoghurt. The ranges of 1.4-4.8 mg/l of intrinsic Zn (II) in milk and 1.2-2.9 mg/kg for yoghurt were determined using commercially available brands. Therefore, it is likely that the biosensors harbour practically available ranges of detection for As (III), Cd (II), and Zn (II).

The GFP-tagged *trans* factors responded to several metals and metalloids. Among these elements, the responses and sensitivities to Sb (III) were lower than those to other toxic metals [13]. Pb (II) or Zn (II) in whole milk and yoghurt bound to the low molecular weight ligands or the milk proteins strongly so that CadC-GFP could not respond to these metals sufficiently. Therefore, specificity to As (III) or Cd (II) in the direct bioassay using whole milk and yoghurt would be expected. It has been reported that, among heterogeneous elements generally contained in milk and yoghurt, Ca (II) and Mg (II) affected fluorescence intensity of ArsR-GFP and CadC-GFP [13]. Therefore, it remains to be clarified before practical use whether the fluorescence intensity is affected by Ca (II) and Mg (II) when As (III) or Cd (II) would be quantified directly using whole milk and yoghurt. It is also important to prepare standard solution or whole product that could control fluorescence intensity as a background.

The toxic metal biosensors have been developed based on interactions between GFP-tagged *trans* factors and immobilized *cis* elements. In addition to the biosensors composed of protein and DNA, a large number of recombinant whole-cell sensors that utilize the sensitivity and selectivity of *trans* factors have been reported. Practical advantages of such biosensor in respect of portability, cost, and manipulation were shown in monitoring drinking and environmental water when it was compared to conventional analytical instruments. However, recombinant whole-cell sensors that could work under nutrient-rich and not defined conditions, as in milk and yoghurt, had not been established. It is expected that whole-cell sensors that can detect Zn (II) in nutrient-poor water might not work in milk and dairy products because carbon sources and other nutrients must affect their metabolisms and cell growth. Even though conventional analytical instruments would be used, extraction techniques of zinc from milk and dairy products will be required prior to measurement as shown previously [9]. Considering such complexity, it is quite advantageous that the elements of biosensor can work in the whole products towards As (III) and Cd (II) as well as in the whey fractions obtained with extraction towards Zn (II). The isolation of *trans* factors and *cis* elements from bacterial cells enabled application of transcriptional switches to sensing toxic metals in the dairy products. To our knowledge, the assays developed in this study are the first one that could quantify toxic metals in milk and yoghurt using biosensing elements.

## Conclusions

Interactions between GFP-tagged *trans* factor and *cis* element could be effectively applied to measurement of As (III), Cd (II), and Zn (II) in milk or yoghurt. Fluorescence intensities obtained with the separately prepared biosensors, which arose from the associated protein, decreased significantly with increasing concentrations of the toxic metals, whereas fluorescence intensities with the solid phase biosensors, which arose from the dissociated protein, increased in response to Zn (II). The GFP-tagged proteins were able to respond to As (III) within ranges of 10–100 μg/l in milk and 10–100 μg/kg in yoghurt and Cd (II) within ranges of 5–100 μg/l in milk and 5–100 μg/kg in yoghurt. However, lower reduction of fluorescence response was obtained towards Pb (II) and Zn (II). Therefore, the optimized pre-treatments, which were lowering pH and 100-times dilution of the obtained whey fractions, were important for measuring Zn (II) in milk and yoghurt. Positive correlations were found between Zn (II) determined with the separately prepared biosensor or solid phase biosensor and total zinc determined with ICP-AES. Thus, the interaction between *trans* factor and *cis* element could be utilized to simple quantification of toxic metals in milk and yoghurt that protects us from excessive and chronic exposure to them.

## Methods

### Preparation of cell lysates containing GFP-tagged *trans* factor

An *arsR* gene encoding the As (III)-binding regulatory protein originated from *Escherichia coli* K12 DNA and a *cadC* gene encoding the Cd (II)/Pb (II)/Zn (II)-binding regulatory protein from *Staphylococcus aureus* NCTC50581 plasmid pI258 have been fused to a structural gene for the green fluorescent protein (AcGFP1) to produce ArsR-GFP and CadC-GFP, as described previously [13]. Recombinant *E. coli* strains were grown in Luria–Bertani (LB) medium supplemented with ampicillin (50 μg/ml) and chloramphenicol (34 μg/ml) at 25°C for 24 h with 120 rpm in reciprocating shaker. Cell lysate containing either ArsR-GFP or CadC-GFP was prepared from the cultures containing 2 × 10^9^ cells/ml as described previously [13].

### Preparation and immobilization of promoter−operator DNA

The double-stranded *ars* promoter−*ars* operator, *P*_ars_−*O*_ars_, and the *cad* promoter−*cad* operator, *P*_cad_−*O*_cad_, were prepared by mixing either *P*_ars_−*O*_ars_-50 or *P*_cad_−*O*_cad_-50, whose 3^′^ end was modified with biotin, and their complimentary oligonucleotide [13] at 50 μM, denaturing at 94°C for 2 min, and cooling down to room temperature. The double-stranded DNA fragments in 25 mM Tris–HCl buffer pH7.4 were immobilized at a concentration of 30 pmol/50 μl *P*_ars_−*O*_ars,_ or 25 pmol/50 μl *P*_cad_−*O*_cad_ onto a Reacti-bind streptavidin-coated high binding capacity black 96-well microplate (Thermo Fisher Scientific, Yokohama, Japan) as described previously [13]. After the incubation, excess unbound DNA was rinsed off 3 times by 25 mM Tris–HCl (pH7.4) buffer.

### Addition of toxic metals to milk and yoghurt

**Figure 6:**
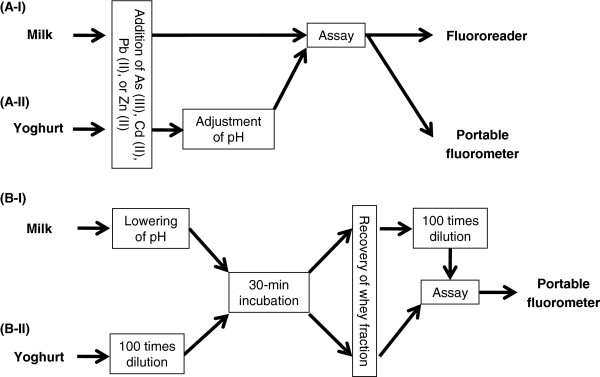
**Schematic representation of milk and yoghurt sample preparation for bioassays.** Preparation of milk (**A**-**I**) and yoghurt (**A**-**II**) that includes externally added toxic metal for bioassays with whole products. Pre-treatments for milk (**B**-**I**) and yoghurt (**B**-**II**) to prepare their whey fractions for Zn (II) bioassays.

Standard solutions of As (III), Cd (II), Zn (II), and Pb (II) were prepared by dissolving NaAsO_2_, CdCl_2_·2.5H_2_O (both from Sigma-Aldrich, Tokyo, Japan), ZnSO_4_·7H_2_O, and Pb(C_2_H_3_O_2_)_2_·3H_2_O (both from Wako Pure Chemical, Osaka, Japan) in ultrapure water (Simplicity UV, Millipore-Japan, Tokyo). Milk and yoghurt were collected from local supermarket at Utsunomiya, Japan and stored at 7°C. Milk was fortified with 5, 10, 50, and 100 μg/l of As (III), Cd (II), Pb (II), or Zn (II) and homogenized properly (Figure 6A). Yoghurt was also fortified with the same concentrations per kg, and then, pH of yoghurt sample was adjusted to 7.0 ± 0.2 before assay because yoghurt itself is acidic and it hampers biosensing.

### Pre-treatments of milk and yoghurt to prepare whey fractions for Zn measurement

For milk, 10.0 ml was sampled and its pH was lowered to 4.6 or below (Figure 6). For yoghurt, 0.50 g was taken and diluted 100 times with ultrapure water. Then, the pH lowered milk and diluted yoghurt were incubated for 30 min to allow zinc shift from curd to whey and finally centrifuged for 30 min at 4°C and 10,000 × *g*. The whey fractions were collected in sterile tubes and used for biosensing. Prior to biosensing, the whey fractions obtained from milk were diluted 100 times to decrease Zn (II) concentration.

### Measurement of arsenic and cadmium by AAS and zinc by ICP-AES

All glassware and crucibles were cleaned by soaking with 0.5 N HNO_3_ overnight and rinsed several times with deionized water and dried prior to use.

For arsenic and cadmium measurement by ET-AAS, milk and yoghurt were digested by microwave-induced combustion methods. Whole milk and yoghurt were fortified with 5, 10, 20, 50, and 100 μg/l of As (III) or Cd (II). After homogenization, 5.00 ± 0.01 g of milk or yoghurt was weighed and poured in teflon microwave digestion vessel. Five milliliters of 50% (v/v) HNO_3_ and 2 ml 30% (v/v) H_2_O_2_ were added to the sample mixtures in digestion vessels. The vessels were closed and fastened in the rotor, and placed into the microwave-induced digestion device (ETHOS-900, Milestone-general, Kawasaki, Japan). A microwave digestion program applied was 250 W for 1 min, 0 W for 1 min, 250 W for 5 min, 400 W for 5 min, and 650 W for 5 min. After digestion, all the mixtures were evaporated. The dried residues were recovered by 5 ml each of 0.5 N HCl. The concentrations of As (III) and Cd (II) were determined by ET-AAS (Z-5010, Hitachi high-technologies, Tokyo, Japan).

An ashing aid suspension was prepared at concentrations of 100 g/l Mg(NO_3_)_2_·6H_2_O and 10 g/l MgO (both from Sigma-Aldrich) using ultrapure water. After homogenization, 5.00 ± 0.01 g of whole milk or yoghurt was weighed and poured into a porcelain crucible for zinc measurement. The milk or yoghurt portion was homogenized again after adding 0.25 ml of the ashing aid suspension to improve decomposition of the organic matrix, and evaporated to near dryness at 105°C in an oven. Two milliliters each of 50% (v/v) HNO_3_ was added to the oven dried residues and the mixtures were dried off on a hotplate. Then, the residues were heated in an electric muffle furnace (FUW 220PA, Advantec, Tokyo, Japan) at atmospheric pressure using the following heating program: 150°C for 1 h, 200°C for 2 h, 250°C for 1 h, 300°C for 3 h, 350°C for 30 min, 400°C for 30 min, and finally 450°C for 14 h [20]. One milliliter each of 50% (v/v) HNO_3_ was added to the grey residues in crucibles and the mixtures were dried off on a hotplate. Then, the residues were reheated in a muffle furnace using the same temperature-time program as shown above. The crucibles containing white ashes were removed from the muffle furnace and cooled to room temperature. The white ashes were dissolved in 5 ml each of 0.5 N HCl and the solutions in the crucibles were transferred to clean glass tubes. The concentrations of Zn (II) in the solutions were determined by inductively coupled plasma-atomic emission spectrometry (ICP-AES) (ICPS-7500, Shimadzu, Kyoto, Japan). Measurement was repeated 3 times for each sample.

### Quantification of metals by the separately prepared biosensors

The separately prepared biosensors, which were based on interaction between CadC-GFP and *P*_cad_−*O*_cad_ or between ArsR-GFP and *P*_ars_−*O*_ars_, were applied to measurements of externally added As (III), Cd (II), Pb (II), and Zn (II) in whole milk or yoghurt. For the As (III) assay, 93 volumes of whole milk/yoghurt or whey fraction was mixed with 5 volumes of 1 M potassium phosphate buffer (KPB) pH6.7, 0.5 volumes of 10 mg/ml salmon sperm DNA, 1 volume of 4 M NaCl, and 0.5 volumes of ArsR-GFP (final concentrations; 50 mM KPB pH6.7, 50 μg/ml salmon sperm DNA, 40 mM NaCl, approximately 20 μg/ml ArsR-GFP). For the Cd (II), Pb (II), and Zn (II) assay, the milk/yoghurt or whey fraction was mixed with the same composition except for that 1 M Tris–HCl pH7.4 and CadC-GFP were used instead of 1 M KPB pH6.7 and ArsR-GFP. The ArsR-GFP or CadC-GFP mixture was pre-incubated at room temperature for 15 min and 100 μl aliquots were poured on the wells of microplate, on which *P*_cad_−*O*_cad_ or *P*_ars_−*O*_ars_ was immobilized. Then, the microplate was incubated for 15 min with orbital shaking at 120 rpm, and supernatants were removed from the wells. The wells were once washed off with 200 μl KP-T buffer (10 mM potassium phosphate pH6.0, 0.05% (wt/vol) Tween20), and 150 μl of measuring buffer (20 mM Tris–HCl pH7.9, 1.0 M NaCl, and 0.10% (wt/vol) Tween20) was added. After incubation for 5 min to dissociate proteins from the surface of wells, the supernatants were transferred to wells of another black plate or glass vessels. Fluorescence in the wells was measured with a microplate fluororeader (MTP-601, Hitachi High Technologies, Tokyo, Japan) at excitation/emission wavelengths of 490/530 nm. A glass vessel prepared from the assay was inserted to an excitation/detection hole of a handheld, battery-powered portable fluorometer (GFP-pen GFP 100, Photon systems instruments, Brno, Czech Republic). After insertion of the vessel, the hole was shaded by a black polyurethane closure and fluorescence from the supernatant was measured. Measurement was repeated three times per vessel. Student’s *t*-test was used to evaluate probability within two groups including data obtained with ultrapure water. Metal concentrations were plotted on a logarithmic scale against fluorescence intensities to evaluate linearity of florescence response.

### Quantification of Zn (II) by the solid phase biosensor

Zn (II) in the prepared whey could be quantified with the solid phase biosensor. In this assay, the number of steps was reduced by directly adding whey fractions to the wells whose surface was modified with a complex of CadC-GFP and immobilized *P*_cad_−*O*_cad_[14]. The CadC-GFP mixture was prepared at final concentrations of 50 mM Tris–HCl buffer pH7.4, 50 μg/ml salmon sperm DNA, 40 mM NaCl, and approximately 20 μg/ml CadC-GFP. Then, 100 μl of the CadC-GFP mixture was poured to each well, in which *P*_cad_−*O*_cad_ was immobilized, and allowed 15-min incubation. The wells were once washed off with 200 μl KP-T buffer. For Zn (II) assay, 93.5 volumes of prepared whey fraction was mixed with 5 volumes of 1 M Tris–HCl pH7.9, 0.5 volumes of 10 mg/ml salmon sperm DNA and 1 volume of 4 M NaCl (final concentrations; 50 mM Tris–HCl, 50 μg/ml salmon sperm DNA, 40 mM NaCl). One hundred five microliters of sample mixture were added to each well, and incubated for 15 min with orbital shaking at 120 rpm to make Zn (II)-bound CadC-GFP release from immobilized *P*_cad_−*O*_cad_. Fluorescence of CadC-GFP was measured as described above. Zn (II) concentrations in standard solution were plotted on a logarithmic scale against fluorescence intensities to make a standard curve. Zn (II) concentrations in milk and yoghurt were determined by using the standard curves and multiplying the dilution factor.

## Competing interests

The authors declare that they have no competing interests.

## Authors’ contributions

MSRS performed all data acquisition, data analyses, and manuscript writing. IM and SU contributed to conception of the study, experimental design, and revision of manuscript. The manuscript was finally read and approved by all co-authors to be published.

## Supplementary Material

Additional file 1 Reproducibility of bioassays using the separately prepared biosensors for milk (A and B) and yoghurt (C and D). Click here for file

Additional file 2 **Fluorescence data in bioassays for the whey fractions prepared from different brands of milk (a-c) and yoghurt (d-g) using the separately prepared biosensor (CadC-GFP and*P***_**cad**_ − ***O***_**cad**_**) and fluorometer.** Click here for file

Additional file 3 **Fluorescence data in bioassays for the whey fractions prepared from different brands of milk (a-c) and yoghurt (d and e) using the solid phase biosensor (CadC-GFP and*P***_**cad**_ − ***O***_**cad**_**) and fluorometer.** Click here for file
