# Supplementary material for: Fluorescent bioassays for toxic metals in milk and yoghurt
Source: BMC Biotechnol. 2012 Oct 25;12:76. doi: 10.1186/1472-6750-12-76 (PMC3505735; doi:10.1186/1472-6750-12-76)
Supplement: Additional file 1 — Reproducibility of bioassays using the separately prepared biosensors for milk (A and B) and yoghurt (C and D). [file 1472-6750-12-76-S1.docx]

**Fluorescent bioassays for toxic metals in milk and yoghurt**

**M. S. R. Siddiki,^1^ S. Ueda,^1,2^ and I. Maeda^1,2^**

^1^United Graduate School of Agricultural Science, Tokyo University of Agriculture and Technology, 3-5-8 Saiwaicho, Fuchu 183-8509, Japan

^2^Faculty of Agriculture, Utsunomiya University, 350 Minemachi, Utsunomiya 321-8505, Japan.

### Reproducibility of bioassays using the separately prepared biosensors for milk (A and B) and yoghurt (C and D)

Fluorescence values measured by fluorometer arose from ArsR-GFP associated with *P*_ars_−*O*_ars_ and CadC-GFP associated with *P*_cad_−*O*_cad_ after incubation with As (III) (A and C) and Cd (II) (B and D), respectively. A solid line and two broken lines show a mean ± SD of data obtained with milk or yoghurt without addition of As (III)/Cd (II). Asterisk means statistically significance versus the milk or yoghurt without addition of As (III)/Cd (II) (**P* < 0.05, ***P* < 0.01, ****P <* 0.001).
