# Supplementary material for: Fluorescent bioassays for toxic metals in milk and yoghurt
Source: BMC Biotechnol. 2012 Oct 25;12:76. doi: 10.1186/1472-6750-12-76 (PMC3505735; doi:10.1186/1472-6750-12-76)
Supplement: Additional file 3 — Fluorescence data in bioassays for the whey fractions prepared from different brands of milk (a-c) and yoghurt (d and e) using the solid phase biosensor (CadC-GFP andPcad − Ocad) and fluorometer. [file 1472-6750-12-76-S3.docx]

**Fluorescent bioassays for toxic metals in milk and yoghurt**

**M. S. R. Siddiki,^1^ S. Ueda,^1,2^ and I. Maeda^1,2^**

^1^United Graduate School of Agricultural Science, Tokyo University of Agriculture and Technology, 3-5-8 Saiwaicho, Fuchu 183-8509, Japan

^2^Faculty of Agriculture, Utsunomiya University, 350 Minemachi, Utsunomiya 321-8505, Japan.

### Fluorescence data in bioassays for the whey fractions prepared from different brands of milk (a-c) and yoghurt (d and e) using the solid phase biosensor (CadC-GFP and *P*_cad_−*O*_cad_) and fluorometer

In each assay, the Zn (II) standard solutions were included.
